# Detection of Schizophrenia Cases From Healthy Controls With Combination of Neurocognitive and Electrophysiological Features

**DOI:** 10.3389/fpsyt.2022.810362

**Published:** 2022-04-05

**Authors:** Qing Tian, Ning-Bo Yang, Yu Fan, Fang Dong, Qi-Jing Bo, Fu-Chun Zhou, Ji-Cong Zhang, Liang Li, Guang-Zhong Yin, Chuan-Yue Wang, Ming Fan

**Affiliations:** ^1^Laboratory of Brain Disorders, Collaborative Innovation Center for Brain Disorders, Beijing Institute of Brain Disorders, Capital Medical University, Ministry of Science and Technology, Beijing, China; ^2^Suzhou Guangji Hospital, The Affiliated Guangji Hospital of Soochow University, The Institute of Mental Health, Suzhou, China; ^3^Beijing Key Laboratory of Mental Disorders, The National Clinical Research Center for Mental Disorders, Beijing Anding Hospital, Beijing Institute for Brain Disorders Center of Schizophrenia, Capital Medical University, Beijing, China; ^4^Department of Psychiatry, First Affiliated Hospital of Henan University of Science and Technology, Luoyang, China; ^5^Beijing Advanced Innovation Centre for Biomedical Engineering, Beijing Advanced Innovation Center for Big Data-Based Precision Medicine, The School of Biological Science and Medical Engineering, Beihang University, Beijing, China; ^6^Department of Psychology, Peking University, Beijing, China; ^7^Advanced Innovation Center for Human Brain Protection, Capital Medical University, Beijing, China; ^8^Institute of Military Cognition and Brain Sciences, Academy of Military Medical Sciences, Beijing, China

**Keywords:** schizophrenia, neurocognition, electrophysiology, electroencephalography, prepulse inhibition (PPI), biomarker, machine learning, classification

## Abstract

**Background:**

The search for a method that utilizes biomarkers to identify patients with schizophrenia from healthy individuals has occupied researchers for decades. However, no single indicator can be employed to achieve the good in clinical practice. We aim to develop a comprehensive machine learning pipeline based on neurocognitive and electrophysiological combined features for distinguishing schizophrenia patients from healthy people.

**Methods:**

In the present study, 69 patients with schizophrenia and 50 healthy controls participated. Neurocognitive (contains seven specific domains of cognition) and electrophysiological [prepulse inhibition, electroencephalography (EEG) power spectrum, detrended fluctuation analysis, and fractal dimension (FD)] features were collected, all these features were taken together to generate the identification models of schizophrenia by applying logistics, random forest, and extreme gradient boosting algorithm. The classification capabilities of these models were also evaluated.

**Results:**

Both the neurocognitive and electrophysiological feature sets showed a good classification effect with the highest accuracy greater than 85% and AUC greater than 90%. Specifically, the performances of the combined neurocognitive and electrophysiological feature sets achieved the highest accuracy of 93.28% and AUC of 97.91%. The extreme gradient boosting algorithm as a whole presented more stably and precisely in classification efficiency.

**Conclusion:**

The highest classification accuracy of 93.28% by combination of neurocognitive and electrophysiological features shows that both measurements are appropriate indicators to be used in discriminating schizophrenia patients and healthy individuals. Also, among three algorithms, extreme gradient boosting had better classified performances than logistics and random forest algorithms.

## Introduction

Schizophrenia is one of the most severe mental disorders, affecting 20 million people worldwide (1). Extensive studies show that cognitive deficits are one of the core features of significant neurological dysfunction associated with schizophrenia and are typically associated with a poor prognosis (2, 3). In addition, cognitive deficits are no less predictive of schizophrenia and its level than positive and negative symptoms, if not better (4, 5). Several specific areas of cognition can be assessed by a neurocognitive measure battery, which usually involves the speed of processing, attention, working memory, and verbal learning (6).

In addition, pre-pulse inhibition (PPI) is considered an indicator that reflects information processing deficits in patients with schizophrenia, which is based on electrophysiological measures (7, 8). Most previous studies show that PPI is reduced in schizophrenic patients and their unaffected first-degree relatives (9). However, in comparison to neurocognitive measurements, PPI presents only a moderate effect size (Cohen’s d < 0.8) (10). Yang et al. (11) reports a novel PPI paradigm involving attentional enhancement effects of PPI while providing a more significant effect size (Cohen’s d > 1.2). Additionally, electroencephalography (EEG) is a non-invasive electrophysiological measure widely applied to assess the neural response of the brain to external stimulation. The EEG power spectrum describes the distribution of power into each frequency band and is commonly used in schizophrenia research (12, 13). Extensive research shows that patients with chronic schizophrenia have abnormal EEG frequencies at rest compared with healthy individuals (12, 14–16). Meanwhile, more advanced EEG analytical methods have been studied in recent years, such as detrended fluctuation analysis (DFA), a fractal analytical method to quantify long-range temporal correlations (LRTCs) in power-law form. A previous study reports strongly reduced LRTCs in both alpha and beta frequency bands in patients with schizophrenia (17).

The current gold standard for schizophrenia diagnosis is built on the International Classification of Diseases, 11th Revision (ICD-11) or the Diagnostic and Statistical Manual of Mental Disorders, 5th Edition (DSM-5). These diagnostic methods rely on descriptive psychopathology, which, to some extent, reflects the subjective judgment of psychiatrists. Therefore, there is an urgent need for clinicians to have an objective measure of characteristics. Nevertheless, frustratingly, due to the heterogeneity of the etiology and clinical variability, excellent biomarkers for the diagnosis of schizophrenia are still lacking. In fact, there is no single indicator that can be adopted in clinical practice. In recent years, the role of machine learning in auxiliary diagnosis has received increasing attention throughout the field of schizophrenia research. In translational medicine and clinical practices, these methods are widely involved in exploration for presymptomatic screening, prognostic prediction, and supporting treatment decisions (18). However, to date, there is a paucity of literature about building machine learning models on neurocognitive and electrophysiological biomarkers.

This study developed a comprehensive machine learning pipeline based on neurocognitive (contains seven specific areas of cognition) and electrophysiological [PPI, EEG power spectrum, detrended fluctuation analysis, and fractal dimension (FD)] features by using logistics, random forest, and extreme gradient boosting (XGBoost) algorithms and evaluated their classification capabilities separately.

## Materials and Methods

### Experimental Subjects

This study enrolled 69 patients with schizophrenia and 50 healthy controls. The diagnosis was established by the researchers from interviews using the Structured Clinical Interview for DSM-IV (SCID) and supplemented by clinical notes. All subjects were right-handed, and their audiometric assessments (pure tone audiometry, 1,000 Hz) were normal. The inclusion criteria for the enrollment of patients with schizophrenia are as follows: 1) all clinically stable subjects had no history of neurological disorders or head trauma, 2) no history of electroconvulsive therapy within the past 6 months, and 3) no history of alcohol/drug dependence or abuse (except tobacco). Patients were excluded because of unstable medical conditions or IQ below 70. During this study, all patients received antipsychotic treatments as usual. The healthy control group (CON) consisted of subjects matched to the schizophrenia group (SCZ) in terms of gender, age, years of education, and smoking history. The exclusion criteria for the CON include substance abuse, suicidal risk, major head trauma, and neuropsychiatric disorders. Before signing the informed consent, each subject received a detailed description of the aims and procedures for participation in the study. The independent ethics committee of Beijing Anding Hospital approved the study. The psychopathological status of the patients was also assessed by the Positive and Negative Syndrome Scale (PANSS). The demographic and clinical characteristics of SCZ and CON are summarized in Table 1.

**TABLE 1:** Demographic and clinical characteristics of healthy control and schizophrenia group.

Factor	CON (*N* = 50)	SCZ (*n* = 69)	χ^2^/t	*P*
Gender (male/female)	38/12	49/20	0.37	0.545^a^
Age (year)	42.2 ± 8.8	44.8 ± 7.0	−1.80	0.074^b^
Education (years of schooling)	10.9 ± 3.1	10.8 ± 2.5	0.15	0.882^b^
Smoking (yes/no)	24/26	35/34	0.09	0.769^a^
Duration of illness (year)		19.7 ± 8.3		
Age at onset (year)		24.5 ± 6.6		
CPZe (mg/day)		292.7 ± 265.3		
PANSS score		63.3 ± 13.1		
Positive Symptoms		12.8 ± 4.5		
Negative Symptoms		20.0 ± 6.4		
General Psychopathology		30.5 ± 5.6		

*Mean ± SD are reported for age, education, duration of illness, age at onset, CPZe, and all PANSS scores.*

*CON, Health Control Group; SCZ, Schizophrenia Group; CPZe, Chlorpromazine Equivalent Doses; PANSS, Positive and Negative Syndrome Scale.*

*^a^Indicates P-value for chi-square test.*

*^b^Indicates P-value for independent sample t-test.*

### Neurocognitive Assessments

The neurocognitive function of the subjects was assessed using the Repeatable Battery for the Assessment of Neuropsychological Status (RBANS; Chinese version) (19). The RBANS assesses five separate cognitive domains: immediate memory (IMM), delayed memory (DEM), visuospatial and constructional (VC), attention (ATT), and language functioning (LAN). Besides RBANS, the Stroop Color-Word Test (Chinese version) (20) was also administered. Each subject was asked to complete two interference tasks, and color (INT-C) and word (INT-W) interference times were recorded. In this part of the experiment, five RBANS features (IMM, DEM, VC, ATT, LAN) and two Stroop features (INT-C, INT-W) were extracted.

### Electrophysiological Assessments

#### Prepulse Inhibition Measures

Subjects were comfortably seated in a reclining chair with their arms fully relaxed in a natural position. Acoustic startle measured through electromyogram (EMG) signal was recorded from the right orbiculate oculi muscle. Electrode impedances were maintained at <5 kΩ. The eye-blink component of auditory startle reflexes was quantified by the human EMG startle reflexes system (EMG XEYE human startle reflex, Tian Ming Hong Yuan Instruments Company, Beijing, China). In addition, the EMG was bandpass filtered to 100–1,000 Hz and amplified 10,000 times. Acoustic startle stimuli were presented binaurally through two headphones. Acoustic signals were characterized by a sound-level meter (AUDit and System 824, Larson Davis, United States).

PPI was tested according to the same paradigm used in previous research (11, 21). In short, the precedence effect was utilized to generate two different perceived spatial relations between the prepulse and background sound: perceptual separation and perceptual colocation. A more detailed description of the PPI paradigm and related theories are available in previous studies (11, 21). Finally, two features were extracted from this test (perceived spatial colocation PPI, PSC-PPI; perceived spatial separation PPI, PSS-PPI).

#### Electroencephalography Recording and Processing

##### Electroencephalography Data Preprocessing

Subjects were comfortably seated in a reclining chair. Then, they closed their eyes and remained relaxed and quiet for 5 min. Continuous EEG was digitized at 1,000 Hz using the EGI EEG system (EGI, Electrical Geodesics, Inc., America) with 128-electrode HydroCelnet referenced to the vertex (Cz). Off-line preprocessing of EEG data was conducted by using EEGLAB (v2019.1) (22) and FieldTrip (23) toolboxes in MATLAB (MATLAB Release 2017b, MathWorks, Inc.). EEG raw data was first resampled to a 500-Hz sampling rate and bandpass filtered to 0.5–45 Hz. For each subject, artifact removal was administered using both continuous raw data and independent component analysis (ICA, algorithm: runica) within EEGLAB. ICA components were classified using an EEGLAB plugin *ICLabel* tool (24). Eye movement, blink, heartbeat, muscular activity, or other artifacts were distinguished from the ICA data. The EEG data were then manually inspected to verify artifact removal. The bad electrodes were replaced with interpolated data from the remaining electrodes. Finally, all electrodes were rereferenced to an average reference.

##### Power Spectrum Features

The power spectral density (PSD) of each electrode was evaluated using the Fast Fourier Transform (FFT, Welch method, 2s sliding window, 50% overlap, 0.5-Hz frequency step), yielding an EEG spectrum ranging from 0.5 to 45 Hz. The frequency bands were selected as follows: delta (1.0–4.0 Hz), theta (4.0–8.0 Hz), alpha (8.0–14.0 Hz), beta (14.0–30.0 Hz). D, T, A, and B denote delta, theta, alpha, and beta frequency bands, respectively. AL and AR were computed by averaging the power in the alpha band for the left (Fp1, F3, C3, P3, O1, F7, T3, T5) and right hemispheres (Fp2, F4, C4, P4, O2, F8, T4, T6). (D + T)L and (D + T)R were averaged by summing the power of delta and theta in the left and right hemispheres. AFp and AO were calculated by averaging the alpha band power for the Fp channels (Fp1, Fp2) and the O channels (O1, O2). The absolute power (Abs) and relative power (Rel) in each frequency band were computed for each electrode. A/T ratio, A/B ratio, (D + T)/(A + B) ratio, (D + T)/(A + B) ratio, (D + T)L/(D + T)R ratio, AFp/AO ratio were calculated for Abs as well as Rel. In PSD, 20 features were extracted. For detailed features information, see Table 2.

**TABLE 2:** Statistical comparison of neurocognitive and electrophysiological features.

Features	CON (*N* = 50)	SCZ (*n* = 69)	t	*P* ^a^
IMM ***	89.24 ± 19.73	54.70 ± 15.30	10.75	0.000
VC **	89.92 ± 20.65	78.81 ± 15.47	3.36	0.003
LAN ***	91.88 ± 16.30	77.68 ± 12.51	5.38	0.000
ATT ***	104.34 ± 15.94	91.09 ± 12.26	5.13	0.000
DEM ***	90.66 ± 19.16	64.39 ± 18.32	7.57	0.000
INT-C **	4.21 ± 3.88	7.39 ± 5.95	–3.30	0.003
INT-W **	16.78 ± 9.27	24.69 ± 12.85	–3.71	0.001
PSC-PPI (%) **	31.70 ± 26.15	11.26 ± 29.07	3.95	0.001
PSS-PPI (%) ***	50.65 ± 25.92	14.70 ± 25.30	7.57	0.000
Abs-D (μV^2^)	10.43 ± 12.57	11.01 ± 13.61	–0.23	0.824
Abs-T (μV^2^) *	3.30 ± 3.60	6.67 ± 7.96	–2.79	0.014
Abs-A (μV^2^)	7.19 ± 8.18	9.78 ± 10.56	–1.45	0.264
Abs-B (μV^2^)	0.80 ± 0.73	0.99 ± 1.07	–1.09	0.376
Abs-A/T	2.79 ± 3.25	2.25 ± 2.41	1.03	0.393
Abs-A/B	9.17 ± 7.99	10.77 ± 9.32	–0.98	0.397
Abs-(D + T)/(A + B)	3.67 ± 6.58	2.27 ± 2.55	1.61	0.202
Abs-(D + T)L/(D + T)R	1.00 ± 0.26	1.05 ± 0.33	–1.01	0.393
Abs-AL/AR	0.98 ± 0.33	0.97 ± 0.28	0.22	0.824
Abs-AFp/AO **	1.49 ± 1.75	0.69 ± 0.61	3.51	0.002
Rel-D *	3.64 ± 1.54	2.98 ± 1.31	2.53	0.028
Rel-T *	1.37 ± 0.65	1.85 ± 1.01	–2.94	0.010
Rel-A	2.32 ± 1.16	2.55 ± 0.89	–1.22	0.376
Rel-B	0.36 ± 0.15	0.34 ± 0.18	0.72	0.550
Rel-A/T	2.46 ± 2.45	2.01 ± 1.82	1.14	0.376
Rel-A/B	8.35 ± 6.62	9.71 ± 6.61	–1.11	0.376
Rel-(D + T)/(A + B)	2.69 ± 2.53	2.18 ± 2.36	1.13	0.376
Rel-(D + T)L/(D + T)R	1.02 ± 0.13	1.05 ± 0.14	–1.10	0.376
Rel-AL/AR	0.98 ± 0.13	0.98 ± 0.12	0.37	0.754
Rel-AFp/AO	0.76 ± 0.42	0.80 ± 0.24	–0.61	0.592
DFA-D	0.73 ± 0.04	0.75 ± 0.07	–2.06	0.082
DFA-T *	0.68 ± 0.05	0.70 ± 0.06	–2.38	0.039
DFA-A **	0.77 ± 0.09	0.71 ± 0.09	3.42	0.003
DFA-B ***	0.66 ± 0.07	0.61 ± 0.06	4.21	0.000
FD	1.60 ± 0.04	1.61 ± 0.04	–0.70	0.550

*Mean ± SD are reported for all features.*

*^a^Indicates P-value for independent sample t-test, and false discovery rate (FDR) was used to adjust P-value.*

*CON, Health Control Group; SCZ, Schizophrenia Group; IMM, immediate memory score; VC, visuospatial/constructional score; LAN, language score; ATT, attention score; DEM, delayed memory score; INT-C, color interference time; INT-W, word interference time; PPI, prepulse inhibition; PSC-PPI, perceived spatial co-location PPI; PSS-PPI, perceived spatial separation PPI; Abs, absolute power spectra; Rel, relative power spectra; D, T, A, B denote delta, theta, alpha, and beta frequency band, respectively; L, left; R, right; Fp, frontal pole; O, occipital; DFA, detrended fluctuation analysis; FD, fractal dimension.*

*P < 0.05; **P < 0.01; ***P < 0.001.

##### Detrended Fluctuation Analysis Features

Detrended fluctuation analysis is an analytical method based on scale-free theory for estimating long-range temporal correlations (LRTCs) in power-law form (25). That is, if a time series data has a non-random temporal structure with slowly decaying autocorrelations, DFA can quantify the rate of decay of these correlations as indexed by the DFA power-law exponent. Some evidence suggests that the DFA reflects brain maturation and may prove useful as a potential biomarker for the pathophysiology of neurodevelopmental disorders (26). DFA calculation was performed using the Neurophysiological Biomarker Toolbox (NBT).^1^ First, all electrodes were filtered in delta, theta, alpha, and beta oscillations, respectively. Then, the amplitude envelope was generated from each frequency band. Finally, the DFA value for each electrode was estimated per participant and stored for each frequency band separately. DFA-D, DFA-T, DFA-A, DFA-B were computed by averaging all electrodes in delta, theta, alpha, and beta frequency bands.

##### Fractal Dimension Features

Brain complexity can be described as the highly structured temporal structure observed in the EEG signal between pure randomness (e.g., white noise) and the absence of variability (constancy or pure periodicity). The EEGLAB plugin *myFractal*^2^ was used to calculate FD for each electrode. Finally, the FD feature was extracted by averaging the FD value of all electrodes.

### Statistical Analyses

Statistics were performed in RStudio (Version 1.2.5033, RStudio, Inc., Boston, United States) with R software (Version 3.6.3). The demographic and primary clinical data include gender, age, years of education, smoking history, duration of illness, age of illness onset, chlorpromazine equivalent doses, PANSS total score, PANSS positive score, PANSS negative score, and PANSS general psychopathology score. All demographic and clinical variables except gender and smoking history were expressed as means and SDs. The independent *t*-test and the chi-square test were conducted to evaluate potential differences in demographic, clinical variables neurocognitive, and electrophysiological between CON and SCZ. The false discovery rate (FDR) was computed to adjust *P*-values for multiple testing based on the Benjamini-Hochberg method (27). *P* < 0.05 (two-tailed) was considered as indicative of statistical significance. PASS version 11.0 (NCSS, LLC., Kaysville, UT, United States) was used for statistical power calculation.

### Classification

All analyses were carried out using R 3.6.3 software. To choose the optimal features for SCZ and CON classification, the classification ability of each feature was first evaluated. A self-compiled function was used to compute Cohen’s d-values of each feature. Receiver operating characteristic curve (ROC) analysis of each feature was created using the package of pROC in R. ROC values of every feature containing accuracy, sensitivity, specificity, and area under ROC curve (AUC) was used for analyses.

All features were then divided into two different categories: neurocognitive and electrophysiological sets. The *rfe* function (R caret package) was used for feature selection by the multivariate recursive feature elimination method (28). The main idea of multivariate recursive feature elimination is to build the model repeatedly and then select the best (or worst) features, put the selected features aside, and then repeat the process over the remaining features until all the features have been traversed. In this process, the order to be eliminated is the order of the features. The *rfe* was first fitted to all features using the bagged tree algorithm. Each feature was ranked according to its importance to the model. In each iteration of feature selection, the ranked features were retained, the model was refitted, and performance was assessed. The final selection of features in each set was based on 10-fold cross-validation.

Then, two optimal subsets (neurocognitive and electrophysiological selected feature sets) and one combination feature set (containing two subsets) were obtained. After feature selection, the logistics algorithm (R stats package), the random forest algorithm (29) (R randomForest package), and the Extreme Gradient Boosting XGBoost algorithm (30) (R xgboost package) were utilized to estimate the classification models from the three features sets. When dealing with medium-sized structured data or table data, it is generally considered that the algorithm based on decision tree is the best. Random forest is an ensemble method to build decision trees. Intuitively speaking, each decision tree is a classifier, and then, for an input sample, N trees have N classification results. The random forest integrates all the classified voting results and specifies the classification with the most votes as the final output. The classification results of several weak classifiers are voted to form a strong classifier, which is the idea of the random forest. XGBoost is another ensemble machine learning algorithm that uses a decision tree as a weak classifier and then integrates these weak classifiers into a strong classifier. In the process of integration, different weights are usually given according to classification accuracy of weak classifiers. Moreover, after adding weak classifiers, the data is usually reweighed to strengthen the classification. Shortly after the XGBoost was put forward, 17 of the 29 champions in Kaggle Data Challenge 2015 used the XGBoost method, which defeated the neural network method.

Finally, the performance of these models is validated using the 10-fold cross-validation method. The results of the validation were then averaged. The classification performance was evaluated by accuracy, sensitivity, and specificity. Besides this, the performance of each model was also evaluated using ROC curves.

## Results

### Demographics and Clinical Characteristics

SCZ (*N* = 69) and CON (*N* = 50) were well matched for gender, age, years of education, and smoking history. There were no significant differences between SCZ and CON in the distribution of these characteristics (Table 1).

### Statistical Comparisons of All Extracted Features Between Schizophrenia Group and Control Group

The statistical analysis results of all features are presented in Table 2. The means and standard deviations of all features are shown. In total, all neurocognitive features were statistically different between SCZ and CON. It can also be observed that all PPI features differed significantly between the two groups. Among the EEG power spectrum features, only absolute theta power (Abs-T), absolute power AFp/AO ratio (Abs-AFp/AO), relative delta power (Rel-D), and relative power theta (Rel-T) showed statistically significant differences. In addition, the DFA of CON is lower than that of SCZ (DFA-T) in the theta band as well as both alpha and beta bands of CON being significantly higher than that of SCZ (DFA-A, DFA-B). None of the FDs differed significantly between SCZ and CON. Among all features, the *P*-values for IMM, LAN, ATT, DEM, PSS-PPI, and DFA-B were less than 0.001.

### Cohen’s d and Classification Performance of Single Feature

To choose optimal features to distinguish SCZ from CON, the Cohen’s d and the ROC values were first evaluated for each feature. The ROC values included accuracy (%), sensitivity (%), specificity (%), and AUC (%). Table 3 shows these indexes from the neurocognitive and electrophysiological features. As can be seen in Table 3, a total of 14 features had d-values that exceeded the range of medium effect sizes (Cohen’s d = 0.5) (31), which is generally consistent with the statistical result. These include IMM, VC, LAN, ATT, DEM, INT-C, INT-W, PSC-PPI, PSS-PPI, Abs-T, Abs-AFp/AO, Rel-T, DFA-A, and DFA-B. Among them, the Cohen’s d-values of LAN, ATT, DEM, and PSS-PPI were all greater than 0.8, indicating a large effect size, whereas the Cohen’s d-value of IMM reached 1.42, suggesting a minimal effect size. In Table 3, it is evident that the neurocognitive features performed better than the electrophysiological features in terms of ROC values. IMM was the most suitable neurocognitive feature for classification with an accuracy of 84.03%, and its AUC reached 91.87%. Five neurocognitive features (IMM, LAN, ATT, DEM, and INT-W) had AUC values greater than 70%, demonstrating functional discrimination capacities of features (32). The PSS-PPI was the best potential electrophysiological feature with an accuracy of 80.67% and an AUC of 84.32%. All four electrophysiological features (PSC-PPI, PSS-PPI, DFA-A, and DFA-B) had AUCs greater than 70%.

**TABLE 3:** Cohen’s d and the classification performance of single feature.

Features	Cohen’s d	Accuracy (%)	Sensitivity (%)	Specificity (%)	AUC (%)
IMM	1.42	84.03	73.91	98.00	91.87
VC	0.60	69.75	95.65	34.00	64.65
LAN	0.90	76.47	75.36	78.00	75.74
ATT	0.86	77.31	85.51	66.00	77.84
DEM	1.16	83.19	82.61	84.00	86.58
INT-C	0.59	64.71	53.62	80.00	66.00
INT-W	0.65	67.23	53.62	86.00	71.26
PSC-PPI	0.69	70.59	76.81	62.00	70.61
PSS-PPI	1.16	80.67	79.71	82.00	84.32
Abs-D	0.04	53.78	49.28	60.00	50.00
Abs-T	0.50	67.23	62.32	74.00	67.13
Abs-A	0.27	68.07	86.96	42.00	61.71
Abs-B	0.20	60.5	72.46	44.00	57.88
Abs-A/T	0.19	37.82	7.25	80.00	48.7
Abs-A/B	0.18	67.23	91.3	34.00	58.43
Abs-(D + T)/(A + B)	0.30	63.87	81.16	40.00	56.96
Abs-(D + T)L/(D + T)R	0.19	53.78	23.19	96.00	54.26
Abs-AL/AR	0.04	42.86	43.48	42.00	51.54
Abs-AFp/AO	0.62	68.07	76.81	56.00	67.68
Rel-D	0.46	66.39	82.61	44.00	63.1
Rel-T	0.53	63.03	62.32	64.00	64.35
Rel-A	0.23	63.87	78.26	44.00	56.23
Rel-B	0.13	53.78	36.23	78.00	56.12
Rel-A/T	0.21	56.3	52.17	62.00	52.87
Rel-A/B	0.21	65.55	89.86	32.00	58.35
Rel-(D + T)/(A + B)	0.21	63.03	79.71	40.00	55.59
Rel-(D + T)L/(D + T)R	0.20	58.82	56.52	62.00	56.61
Rel-AL/AR	0.07	47.06	46.38	48.00	47.8
Rel-AFp/AO	0.11	60.5	66.67	52.00	59.13
DFA-D	0.38	57.14	30.43	94.00	58.52
DFA-T	0.43	61.34	62.32	60.00	61.57
DFA-A	0.61	68.07	63.77	74.00	70.1
DFA-B	0.73	71.43	75.36	66.00	71.94
FD	0.13	54.62	46.38	66.00	53.46

*AUC, area under receiver operating characteristic curve; IMM, immediate memory score; VC, visuospatial/constructional score; LAN, language score; ATT, attention score; DEM, delayed memory score; INT-C, color interference time; INT-W, word interference time; PPI, prepulse inhibition; PSC-PPI, perceived spatial co-location PPI; PSS-PPI, perceived spatial separation PPI; Abs, absolute power spectra; Rel, relative power spectra; D, T, A, B denote delta, theta, alpha, and beta frequency band, respectively; L, left; R, right; Fp, frontal pole; O, occipital; DFA, detrended fluctuation analysis; FD, fractal dimension.*

### Classification Performances of Combined Features

As mentioned in the methods, the REF method was used to select the neurocognitive and electrophysiological feature sets that could optimally distinguish between CON and SCZ. In addition, 10-fold cross-validation was used for feature selection to prevent overfitting. Finally, the neurocognitive selected features (NSF) subset contained immediate memory, delayed memory, attention, language functioning, color interference time, and word interference time. The electrophysiological selected features (ESF) subset contained PSC-PPI, PSS-PPI, Abs-T, Abs-A, Abs-AFp/AO, Abs-(D + T)/(A + B), Rel-D, Rel-T, Rel-A/B, DFA-A, and DFA-B. All selected features (ASF) included two subsets as described above. Then, the logistics, random forest and XGBoost algorithms were implemented to build classification models from the NSF subset, ESF subset, and ASF set. On this basis, these models were evaluated using a 10-fold cross-validation method.

**FIGURE 1:**
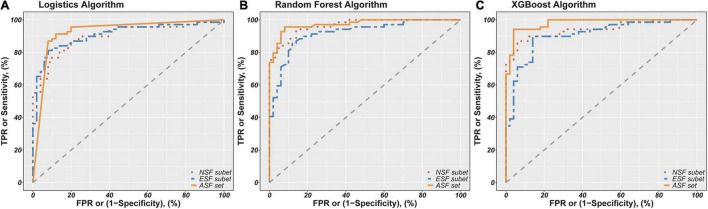
Receiver Operator Characteristics (ROC) curves for classification of schizophrenia patients and controls based on different combinations of features using logistics **(A)**, random forest **(B)**, and XGBoost algorithm **(C)**. NSF subset, Neurocognitive Selected Features subset include IMM, LAN, ATT, DEM, INT-C, INT-W features; ESF subset, Electrophysiological Selected Features subset include PSC-PPI, PSS-PPI, Abs-T, Abs-A, Abs-AFp/AO, Abs-(D+T)/(A+B), Rel-D, Rel-T, Rel-A/B, DFA-A, DFA-B. ASF set, All Selected Features set include NSF subset and ESF subset. The red, green and blue lines show the ROC curves for the NSF subset, ESF subset and ASF set, respectively. The AUC of logistics models based on NSF subset, ESF subset, ASF set was 89.88%, 90.84%, 92.54%. The AUC of random forest models based on NSF subset, ESF subset, ASF set was 96.59 91.88%, 97.36%. The AUC of XGBoost models based on NSF subset, ESF subset, ASF set was 93.99%, 90.52%, 97.91%.

Finally, the fitted values for the probability of people with schizophrenia ranged from 0 to 1. Accuracy, sensitivity, and specificity were calculated by setting cutoff points at 0.5 of the fitted values, and then the ROC curves were evaluated and AUCs were calculated based on the fitted values. These model evaluation indicators (accuracy, sensitivity, specificity, and AUC) are shown in Table 4. Figure 1 shows the ROC curves for each model to represent the differences of ROC curves more clearly. As can be seen from Table 4 and Figure 1, the model containing all selected feature sets exhibits the best classification performance regardless of the algorithm used (logistics accuracy of 87.39%, random forest and XGBoost accuracy of 93.28%). The NSF subset models and the ESF subset models showed roughly comparable levels of classification accuracy, but the NSF subset models performed better than the ESF subset in terms of AUC values. Besides this, the XGBoost algorithms performed more consistently and accurately in both accuracy and AUC among all the compared algorithms. The ASF set model with the XGBoost algorithm achieved the highest accuracy of 93.28% and an AUC of 97.91%. Statistical power was calculated by tests for one ROC curve procedure in PASS software. The power analysis showed that 50 patients and 69 healthy subjects were sufficient to have more than 90% statistical power at a two-sided alpha of 0.05 for significance level (Supplementary Table 1).

**TABLE 4:** Classification performances of combined features.

Feature set	Accuracy (%)	Sensitivity (%)	Specificity (%)	AUC (%)
**NSF subset models**				
Logistics algorithm	82.35	88.41	74.00	89.88
Random forest algorithm	88.24	82.61	96.00	96.59
XGBoost algorithm	89.08	89.86	88.00	93.99
**ESF subset models**				
Logistics algorithm	82.35	86.96	76.00	90.84
Random Forest algorithm	84.87	91.30	76.00	91.88
XGBoost algorithm	88.24	89.86	86.00	90.52
**ASF set models**				
Logistics algorithm	87.39	92.75	80.00	92.54
Random forest algorithm	93.28	94.20	92.00	97.36
XGBoost algorithm	93.28	91.30	96.00	97.91

*NSF subset, Neurocognitive Selected Features subset include IMM, LAN, ATT, DEM, INT-C, INT-W features; ESF subset, Electrophysiological Selected Features subset include PSC-PPI, PSS-PPI, Abs-T, Abs-A, Abs-AFp/AO, Abs-(D + T)/(A + B), Rel-D, Rel-T, Rel-A/B, DFA-A, DFA-B; ASF set, All Selected Features set include NSF subset and ESF subset.*

**FIGURE 2:**
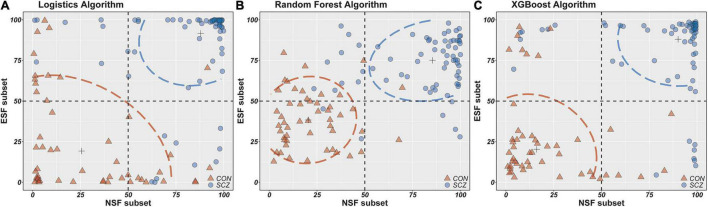
The horizontal/longitudinal coordinate axis represents the probability of people with schizophrenia (%). NSF subset, Neurocognitive Selected Features subset include IMM, LAN, ATT, DEM, INT-C, INT-W features; ESF subset, Electrophysiological Selected Features subset include PSC-PPI, PSS-PPI, Abs-T, Abs-A, Abs-AFp/AO, Abs-(D + T)/(A + B), Rel-D, Rel-T, Rel-A/B, DFA-A, DFA-B; ASF set, All Selected Features set include NSF subset and ESF subset. **(A–C)** Scatter plots from two features set (NSF subset and ESF subset) using logistics, random forest and XGBoost models.

To better present the differences in the classification ability of these three algorithms, scatterplots were drawn with the fitted values of the NSF subset as the horizontal coordinates and the fitted values of the ESF subset as the vertical coordinates (Figure 2). It is apparent that the fitted values of the random forest and XGBoost algorithm are more densely distributed in this plot compared to the logistics algorithm, which also suggests that the random forest and XGBoost algorithms provide better performance in distinguishing schizophrenia patients from healthy individuals.

## Discussion

### Classification Performance of Neurocognition in Schizophrenia

First, this study sought to determine which feature set was more beneficial in distinguishing between schizophrenic patients and healthy control subjects to construct the model. The second aim of the project was to identify which machine learning algorithm performed better in terms of classification ability and robustness.

The highest classification accuracy was 84.03% based on a single neurocognitive feature, achieved by immediate memory. A high level of immediate memory is considered as a predictor of improved cognitive impairments in schizophrenic patients (33). This also implies a potential opportunity for evaluating the prognostic or medical effect by using an immediate memory score. Other neurocognitive indicators performed well. For instance, the delayed memory score also received an excellent classified effect (accuracy of 83.19% and AUC of 86.58%). The classification accuracy of the combined neurocognitive feature set was slightly higher than that of any single feature classifier (accuracy of 89.08% and AUC of 93.99%).

Previous studies demonstrate the importance of neurocognitive-based machine learning techniques for diagnosing schizophrenia. Vacca et al. (34) employed several machine learning techniques (logistics regression, decision tree, random forest, k-nearest neighbor, neural network, support vector machine) to distinguish between 86 schizophrenic patients and 115 healthy subjects. The best methods turned out to be support vector machine and neural network with accuracies of 87 and 84.8%. Antonucci et al. (35) investigated the discriminatory performance of genetic, environmental, and neurocognitive classifiers by using support vector classification and repeated nested cross-validation. The cognitive classifier showed an accuracy of 88.7%, followed by environmental (65.1%) and genetic (55.5%) classifiers. The findings of the two studies are in line with the current study, revealing that neurocognition is a robust indicator in differentiating schizophrenia from healthy people.

### Classification Performance of Electrophysiology in Schizophrenia

PPI deficits are involved in the biological bases of schizophrenia and proposed as a potential biomarker for genetic studies with more than 50% of PPI variance attributed to genetic factors (36). Mackeprang et al. (37) argue that PPI was not affected by antipsychotic treatment and, instead, was a stable vulnerability index for schizophrenia. Mena et al. (38) used longitudinal data to examine whether PPI deficits exhibit a temporary effect in the acute phase of schizophrenia. The result suggests that the PPI remained reduced at the 3 months post-discharge assessment, implying that the PPI was a biomarker of schizophrenia. However, in practice, the PPI did not separate schizophrenic subjects from healthy controls (39). In comparison to neurocognitive and other electrophysiological measures, the PPI showed a medium Cohen’s d-value of below 0.6 (40). The previous study establishes a novel PPI paradigm by using a precedence effect–based perceived separation, which produced a Cohen’s d-value of 1.27 and an AUC of 85.2% (11). The current study analyzes whether PSC-PPI and PSS-PPI single features could distinguish SCZ from CON. The accuracy of PSS-PPI was 80.67%, and the AUC was 84.32%.

In addition, other single markers were tested for their ability to identify schizophrenia based on the EEG power spectrum, detrended fluctuation analysis, and fractal dimension. DFA is a method to evaluate the large-scale functional neural dysconnectivity of schizophrenic patients at the temporal level. It was found that DFA was substantially attenuated in both alpha and beta frequency bands in patients. DFA-B presented the highest accuracy of 71.43% and AUC of 71.94% in a single EEG feature.

Compared with the best single electrophysiological feature (PSS-PPI), the combined electrophysiological feature increased the accuracy from 80.67 to 88.24% and the AUC from 84.42 to 91.88%. Devia et al. (41) report that the EEG signals from a free-viewing paradigm distinguished patients from healthy subjects with an overall accuracy of 71%. Thilakavathi et al. (42) analyzed the EEG power spectrum and found that the vector machine classifier produced an accuracy of 88% when features were combined together. Laton et al. (43) used a combination of oddball and mismatch event-related potentials and increased accuracy from 79.8 to 84.7% for a single feature. The above findings suggest that it is essential to select the appropriate EEG feature set to better distinguish between schizophrenic and healthy individuals.

### Classification Performance of Combined Neurocognitive and Electrophysiological Features in Schizophrenia

The results show that both neurocognitive and electrophysiological feature sets had a good performance with accuracy values greater than 80% and AUC values greater than 85%. Specifically, the combined neurocognitive and electrophysiological features delivered the highest accuracy of 93.28% and AUC of 97.91%. In fact, the XGBoost algorithm as a whole presented a more stable and accurate classification efficiency in this study. As previous research indicates, XGBoost had several advantages in terms of speed and accuracy over other tree-based ensemble methods, such as Random Forests, AdaBoost, and the traditional gradient boosted trees (30).

### Potential Limitations

There are several potential limitations to this study. First, this is a single-center study with limited sample size. Due to the relatively small number of subjects in the study, caution should be exercised when attempting to generalize these findings to clinical applications. Second, there is a lack of drug-naïve patient groups. Drugs may have potentially confounded the findings of the classification performances of these models and clinical status. Third, it is related to the duration of the disease. Nevertheless, no correlation was found between all features and CPZe as well as duration of illness (Table 5). The last one is model interpretability. Indeed, classic statistical regression models, such as linear regression, perform better in terms of interpretability than black-box machine learning models. Perhaps it is for this reason that the acceptance of machine learning among clinicians is lacking. However, schizophrenia is likely to be etiologically heterogeneous, resulting in poor prediction performance of a linear model. Linear regression cannot model the inherent complexity of data sets (such as feature interaction). Therefore, when choosing an appropriate machine learning model, we usually need to weigh the accuracy and interpretability of the model.

**TABLE 5:** Correlation between all features and CPZs, duration of illness in patients.

Features	CPZe		Duration of illness	
		
	r	*P*	r	*P*
IMM	0.100	0.398	–0.050	0.671
VC	0.000	0.991	–0.020	0.861
LAN	–0.100	0.432	0.140	0.245
ATT	–0.090	0.478	0.070	0.563
DEM	0.080	0.518	0.020	0.849
INT-C	–0.010	0.955	–0.010	0.955
INT-W	–0.180	0.132	–0.010	0.926
PSC-PPI	–0.150	0.233	0.080	0.507
PSS-PPI	–0.200	0.101	0.190	0.115
Abs-D	0.050	0.677	0.040	0.768
Abs-T	–0.050	0.708	0.120	0.330
Abs-A	0.050	0.667	0.140	0.264
Abs-B	0.140	0.254	0.130	0.283
Abs-A/T	0.150	0.222	–0.010	0.912
Abs-A/B	–0.050	0.674	0.050	0.705
Abs-(D + T)/(A + B)	–0.090	0.456	–0.010	0.937
Abs-(D + T)L/(D + T)R	–0.010	0.952	0.120	0.321
Abs-AL/AR	–0.080	0.496	0.100	0.421
Abs-AFp/AO	0.030	0.799	–0.010	0.953
Rel-D	0.040	0.763	–0.060	0.610
Rel-T	–0.210	0.078	0.070	0.547
Rel-A	0.080	0.513	0.030	0.803
Rel-B	0.010	0.926	–0.020	0.879
Rel-A/T	0.150	0.211	–0.020	0.893
Rel-A/B	–0.040	0.740	0.070	0.544
Rel-(D + T)/(A + B)	–0.100	0.405	–0.020	0.880
Rel-(D + T)L/(D + T)R	0.130	0.280	0.050	0.677
Rel-AL/AR	–0.170	0.159	0.080	0.519
Rel-AFp/AO	–0.020	0.850	0.090	0.478
DFA-D	0.080	0.503	0.160	0.197
DFA-T	–0.010	0.947	0.080	0.496
DFA-A	–0.040	0.720	–0.040	0.752
DFA-B	0.040	0.740	0.180	0.137
FD	0.130	0.281	–0.080	0.519

*P-value for spearman rank correlation analysis, and false discovery rate (FDR) was used to adjust P-value.*

*CPZe, Chlorpromazine Equivalent Doses; IMM, immediate memory score; VC, visuospatial/constructional score; LAN, language score; ATT, attention score; DEM, delayed memory score; INT-C, color interference time; INT-W, word interference time; PPI, prepulse inhibition; PSC-PPI, perceived spatial co-location PPI; PSS-PPI, perceived spatial separation PPI; Abs, absolute power spectra; Rel, relative power spectra; D, T, A, B denote delta, theta, alpha, and beta frequency band, respectively; L, left; R, right; Fp, frontal pole; O, occipital; DFA, detrended fluctuation analysis; FD, fractal dimension.*

In future research, to provide a simple, robust, and reliable model for detecting schizophrenia, first-episode, drug-naïve patients with schizophrenia will be recruited. Moreover, patients with depression and bipolar disorder will also be invited to participate in a future study to assess the specificity of the model and to determine whether the severity of these features varies across psychiatric disorders. Besides this, additional EEG features may be identified in the classification. Further research may also be conducted to evaluate the efficacy of interventions and prognosis.

## Conclusion

In this study, a comprehensive machine learning pipeline was provided to detect patients with schizophrenia by applying logistics, random forest, and extreme gradient boosting (XGBoost) algorithm classifiers to neurocognition, electrophysiology, and their combination. The highest classification accuracy of 93.28% was achieved by combining neurocognitive and electrophysiological features, suggesting that these measurements are appropriate indicators for discriminating schizophrenia patients from healthy individuals. Also, among these three algorithms, XGBoost has better classification performances than the other two algorithms. These results suggest that neurocognitive and electrophysiological features can be used along with machine learning for potential clinical applications.

## Data Availability Statement

The original contributions presented in the study are included in the article/Supplementary Material, further inquiries can be directed to the corresponding authors.

## Ethics Statement

The studies involving human participants were reviewed and approved by the Ethics Committee of Beijing Anding Hospital. The patients/participants provided their written informed consent to participate in this study.

## Author Contributions

G-ZY, C-YW, and MF designed the study. LL provided the PPI paradigm for the study. QT, N-BY, YF, FD, Q-JB, and F-CZ acquired and analyzed the data. J-CZ supplied the machine learning algorithm for the study. QT wrote the article, which all authors reviewed. All authors approved the final version to be published and can certify that no other individuals not listed as authors have made substantial contributions to the manuscript.

## Conflict of Interest

The authors declare that the research was conducted in the absence of any commercial or financial relationships that could be construed as a potential conflict of interest.

## Publisher’s Note

All claims expressed in this article are solely those of the authors and do not necessarily represent those of their affiliated organizations, or those of the publisher, the editors and the reviewers. Any product that may be evaluated in this article, or claim that may be made by its manufacturer, is not guaranteed or endorsed by the publisher.

## Footnotes

1
https://github.com/NBT-Analytics/NBTpublic

2
https://github.com/rami-codes/myFractal
