# Supplementary material for: Detection of Schizophrenia Cases From Healthy Controls With Combination of Neurocognitive and Electrophysiological Features
Source: Front Psychiatry. 2022 Apr 5;13:810362. doi: 10.3389/fpsyt.2022.810362 (PMC9016153; doi:10.3389/fpsyt.2022.810362)
Supplement: Supplementary file 1 [file Table_1.DOCX]

| Table S1. Statistical power of all models | | | | |
| --- | --- | --- | --- | --- |
| **Feature Set** | **AUC (%)** | | **Power** |  |
| NSF subset models |  |  |  |  |
| Logistics algorithm | 89.88 |  | 1.00 |  |
| Random forest algorithm | 96.59 |  | 1.00 |  |
| XGBoost algorithm | 93.99 |  | 1.00 |  |
| ESF subset models |  |  |  |  |
| Logistics algorithm | 90.84 |  | 1.00 |  |
| Random Forest algorithm | 91.88 |  | 1.00 |  |
| XGBoost algorithm | 90.52 |  | 1.00 |  |
| ASF set models |  |  |  |  |
| Logistics algorithm | 92.54 |  | 1.00 |  |
| Random forest algorithm | 97.36 |  | 1.00 |  |
| XGBoost algorithm | 97.91 |  | 1.00 |  |
| *Note*: NSF subset, Neurocognitive Selected Features subset include IMM, LAN, ATT, DEM, INT-C, INT-W features; ESF subset, Electrophysiological Selected Features subset include PSC-PPI, PSS-PPI, Abs-T, Abs-A, Abs-AFp/AO, Abs-(D+T)/(A+B), Rel-D, Rel-T, Rel-A/B, DFA-A, DFA-B; ASF set, All Selected Features set include NSF subset and ESF subset. | | | | |
