# Supplementary figures and images for: Detection of Schizophrenia Cases From Healthy Controls With Combination of Neurocognitive and Electrophysiological Features
Source: Front Psychiatry. 2022 Apr 5;13:810362. doi: 10.3389/fpsyt.2022.810362 (PMC9016153; doi:10.3389/fpsyt.2022.810362)

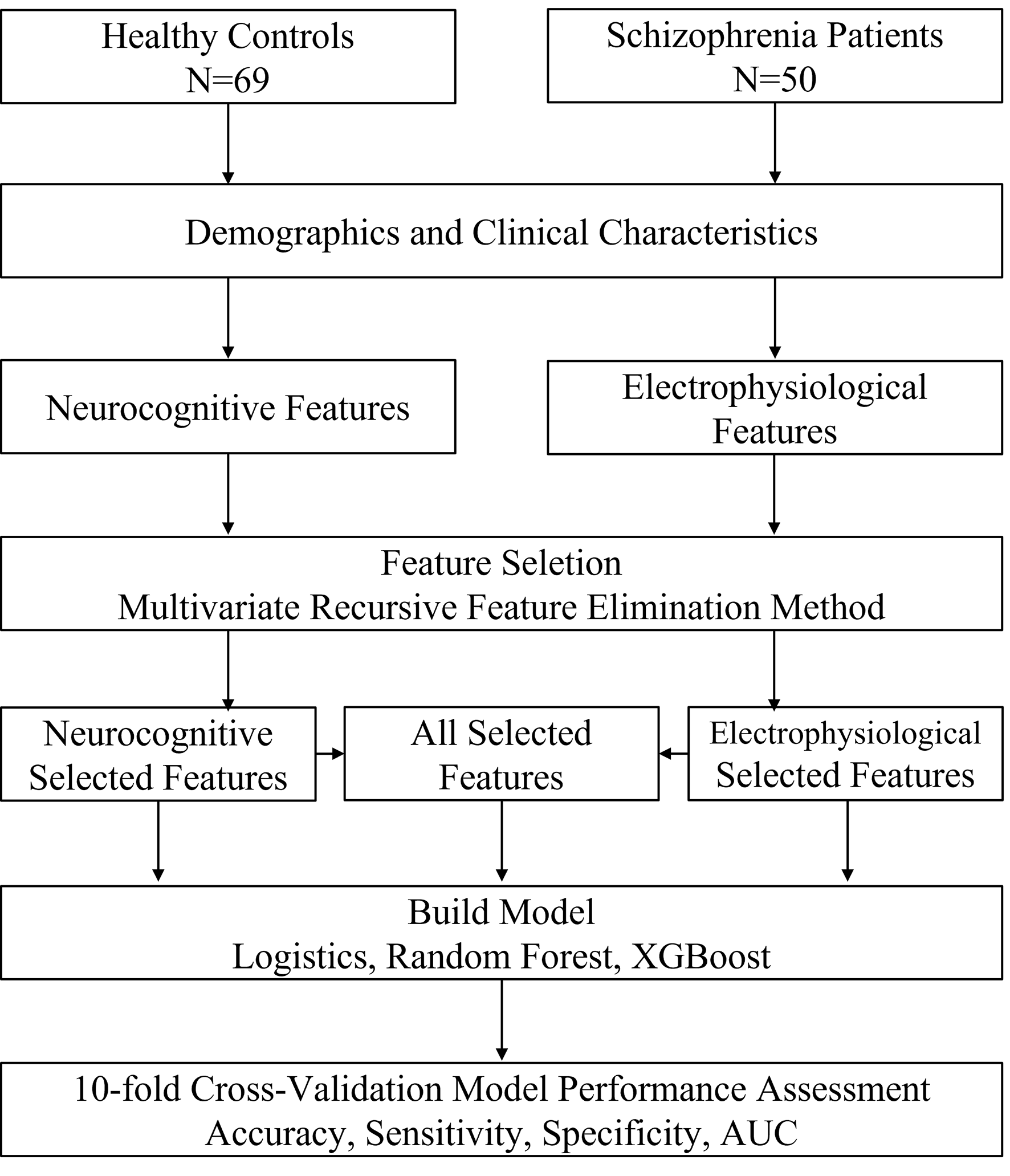

Supplement: Supplementary file 2 [file Image_1.TIF]
